# Cyclitis and Irido-Cyclitis—II

**Published:** 1908-08-01

**Authors:** 


### Ophthalmology.

### CYCLITIS AND IRIDO-CYCLITIS II.

The earlier the diagnosis of iridocyclitis is made the better the chance of saving the eye; for if the pupil can be widely dilated at the very commencement the iris is kept well out of the way, may not become implicated to so great an extent, and leaves the passage between the vitreous and the anterior chambers free for the circulation of fluids, thus lessening the risk of excessive tension. One of the earliest diagnostic signs of cyclitis is œdema of an eyelid, usually the upper, accompanied by neuralgic pain, redness of the conjunctiva, and congestion of the deep vessels. This deeper redness and congestion is usually seen over the site of the ciliary body; that is in a circle round the cornea some 6 mm. wide from the corneo-sclerotic margin. Very frequently a localised patch of œdema of the conjunctiva is seen in this situation. Tenderness on pressure through the eyelid over the site of the ciliary body does not occur if iritis only be present.

One very early sign of cyclitis is the appearance of the uveal pigment in irregular masses at the pupillary margin, or *Ectropion Uveæ*. The appearance of deposits on the back of the cornea, so called keratitis punctata, or K.P., confirms the diagnosis. These deposits in an early stage are frequently impossible to see except with a highly-magnifying corneal lens, or the +20 lens of the ophthalmoscope used in conjunction with reflected light, and for this reason are frequently missed. The deposits are of two kinds: one variety, the earliest to appear, is very small and is composed of cells from the turbid aqueous; the deposits of this kind are found arranged in a triangular shape with the apex of the triangle opposite the centre of the pupil, broadening out to the base at the lower limit of the anterior chamber. They become arranged in this manner owing to centrifugal action, and the oscillatory movements of the eyeball; they at first float freely in the aqueous, for they are continually stired up by the movements of the globe, but after a time begin to stick on the posterior surface of the cornea, the finer ones at the top, the larger and heavier falling as near the lower confines of the anterior chamber as possible. When an excessive amount of exudate is present, it falls to the bottom of the chamber and there forms a hypopyon. These deposits appear to increase in size from day to day, due to new deposit sliding over the smooth posterior surface of the cornea until it comes upon one already adherent, to which in turn it becomes attached; in this way they extend from one deposit to another, become confluent, as it were, and grow into the large deposits designated "mutton-fat like." The other variety of deposit is, in all probability, due to the continuity of Descemet's membrane with the uveal tract, and to inflammatory products creeping along it, and spreading from the circumference towards the centre of the cornea.

The pupil itself, before it shows any of the irregularity caused by iritic adhesions, frequently appears flattened in parts of its curves, and its reactions are also sluggish.

The pain in cyclitis is not only referred to the eyeball itself but radiates in all directions, especially to the supra-orbital region, and becomes neuralgic in type; but pain down the side of the nose is most frequently caused by glaucoma and not by iridocyclitis or cyclitis. This neuralgic pain frequently becomes intolerable, especially at night when the patient has become warm in bed; at times it comes on with extreme rapidity, and it often ceases as suddenly.

Vision becomes diminished early in cyclitis; this is caused by the turbidity of the aqueous and the exudate into the vitreous, shown by the presence of vitreous opacities. Vitreous opacities, together with diminution or dimness of vision and an inflamed and tender eye are very suspicious of cyclitis. If the anterior chamber is deeper than normal the above symptoms are almost diagnostic.

The causes of cyclitis are always constitutional, with the exception of those attacks brought on by a perforating injury of the globe itself, or of the other eye. Perforating injuries includes incisions made during intra-ocular operations. The onset may be immediate or remote, the later attacks brought on probably by entanglement of the iris in the scar of the wound or of a portion of the capsule of the lens similarly caught, and the production of ciliary irritation by traction.

Of the constitutional causes, specific disease is by far the commonest. Rheumatism, particularly rheumatism of gonorrhœal origin (the variety which causes rheumatic iritis), must be looked upon as one of the causes of cyclitis; if gout ever gives rise to inflammation of the ciliary body it must be when largely combined with a rheumatic element. Any general septic condition of the blood, puerperal septicæmia, chronic pyæmic conditions, the less active forms of septic infection from decayed teeth, obstinate constipation, and those conditions known as auto-intoxication or infection, all these give rise to acute cyclitis at times, more usually to chronic cyclitis without any accompanying iritis. Neoplasms in the choroid or in the ciliary body itself will cause cyclitis from direct irritation, but not as a rule until they become necrotic. Bad corneal ulcers also cause irido-cyclitis, not necessarily through a perforation, but apparently from septic absorption, the focus of infection being in such close vicinity. Cyclitis caused in one eye by a perforating injury is very liable to bring about the same inflammation in the other eye; this, which is known as sympathetic ophthalmia, is usually of a very severe type and frequently more destructive than the original inflammation in the exciting eye.

Tubercle also at times affects the ciliary body, and, as a rule, the iris at the same time; the affection of the iris is characterised by the presence of nodules or more frequently of a single nodule in it. The exudate creeps along Descemet's membrane and obscures the view of the iris, making it exceedingly difficult to see what is taking place in the anterior chamber.

